# Probing the Effect of Young’s Modulus on the Reservoir Regulation Abilities of Dispersed Particle Gels

**DOI:** 10.3390/gels9050402

**Published:** 2023-05-11

**Authors:** Zizhao Wang, Zhixuan Zhu, Tianyu Jiang, Jinming Liu, Yunbo Dong, Yining Wu, Mingwei Zhao, Caili Dai, Lin Li

**Affiliations:** 1 Key Laboratory of Unconventional Oil & Gas Development, China University of Petroleum (East China), Ministry of Education, Qingdao 266580, China; wangzizhao1999@163.com (Z.W.);; 2 Shandong Key Laboratory of Oilfield Chemistry, Department of Petroleum Engineering, China University of Petroleum (East China), Qingdao 266580, China; 3 Research Institute of Oil Production Engineering, PetroChina Daqing Oilfield Limited Company, Daqing 163453, China; 4 Heilongjiang Provincial Key Laboratory of Oil and Gas Reservoir Stimulation, PetroChina Daqing Oilfield Limited Company, Daqing 163453, China

**Keywords:** dispersed particle gel, Young’s modulus, reservoir regulation abilities, reservoir environment, migration ability

## Abstract

The mechanical strength of dispersed particle gels (DPGs), which can be directly characterized by Young’s modulus, is an important parameter affecting reservoir regulation performance. However, the effect of reservoir conditions on the mechanical strength of DPGs, as well as the desired range of mechanical strength for optimum reservoir regulation performance, have not been systematically studied. In this paper, DPG particles with different Young’s moduli were prepared and their corresponding migration performances, profile control capacities and enhanced oil recovery abilities were studied by simulated core experiments. The results showed that with increase in Young’s modulus, the DPG particles exhibited improved performance in profile control as well as enhanced oil recovery. However, only the DPG particles with a modulus range of 0.19–0.762 kPa could achieve both adequate blockage in large pore throats and migration to deep reservoirs through deformation. Considering the material costs, applying DPG particles with moduli within the range of 0.19–0.297 kPa (polymer concentration: 0.25–0.4%; cross-linker concentration: 0.7–0.9%) would ensure optimum reservoir control performance. Direct evidence for the temperature and salt resistance of DPG particles was also obtained. When aged in reservoir conditions below 100 °C and at a salinity of 10 × 10^4^ mg·L^−1^, the Young’s modulus values of the DPG particle systems increased moderately with temperature or salinity, indicating a favorable impact of reservoir conditions on the reservoir regulation abilities of DPG particles. The studies in this paper indicated that the practical reservoir regulation performances of DPGs can be improved by adjusting the mechanical strength, providing basic theoretical guidance for the application of DPGs in efficient oilfield development.

## 1. Introduction

Many discovered oil and gas reservoirs belong to continental sedimentary strata with strong heterogeneity; as a result, water injection and flooding has become the most commonly used development method [1,2,3,4,5,6]. With long-term water injection development, injected water has formed dominant seepage channels by crossflow along big pore throats and high-permeability zones [7,8,9]. Due to the permeability differences among reservoir layers, which has been called “reservoir heterogeneity”, working fluid may channel through high-permeability fractures, resulting in low sweep efficiency in low-permeability areas. Therefore, it is necessary to regulate the heterogeneity of reservoirs. A large amount of remaining oil cannot be effectively produced. In order to alleviate the effects of differences between layers, it is necessary to regulate the heterogeneity of reservoirs [10,11]. The means of reservoir regulation include the use of bulk gels, particles (pre-crosslinked particles, polymer microspheres and dispersed particle gel systems), etc. [12,13,14,15,16,17,18,19]. Bulk gels reduce the permeability of large pore channels by cross-linking underground to form blockages to plug formations, but they are susceptible to formation shearing actions [20,21]. Therefore, granular profile control and water shutoff agents were introduced. Pre-crosslinked particle systems showed good water-swelling properties; however, they were likely to be trapped near wellbores due to the relatively large particle sizes [22]. Polymer microspheres have been used to regulate reservoirs through the repeated plugging of pore throats and multiple deformation migration, but their production costs are high due to harsh synthesis conditions [23,24]. Dispersed particle gel (DPG) systems, by contrast, which are prepared by mechanical shearing of bulk gels on the ground, possess the advantages of controllable particle size and low cost. DPG particles can build bridges in pore throats to achieve proper plugging and then regulate deep reservoirs through deformation and migration [12,25,26,27]. DPG particles have enjoyed wide application in deep reservoir profile control.

The properties of DPG particle systems, such as particle size and mechanical strength, have a great influence on reservoir regulation effects. Previous research has been mainly focused on particle size [22,28,29]. The particle size of a DPG particle system must match the size of the pore throats of the relevant formation, so that the particles can be injected into the formation smoothly and plug in the deep zones [29]. Only a suitable particle size could endow the system with good formation adaptability and achieve the purpose of deep reservoir profile control and flooding. Moreover, the aging process has an effect on the particle size of DPG particle systems [30,31]. During the aging process, the particle size of single particles increases due to swelling; the aggregation and adhesion of multiple particles to each other further leads to a significant increase in the average particle size of DPG particles. The mechanical strength of a gel material is a crucial physical property parameter, and its Young’s modulus at the macro-scale is typically measured using a rheometer, which calculates the Young’s modulus of the gel by measuring the energy and loss during the process of shear deformation [32,33,34]. However, due to the unique characteristics of DPGs, conventional methods cannot effectively characterize the physical properties of materials in a liquid environment, rendering them unsuitable for measuring the Young’s moduli of small-scale materials. Previous studies have shown that the mechanical strength of small-scale microspheres can be measured through real-time imaging of samples and the capture of force curves using atomic force microscopy [35,36,37]. Up to now, a standardized method for measuring the Young’s moduli of small-scale gel dispersion particles has not been reported. Therefore, this study provides the first quantitative characterization of the mechanical strength of DPG particle systems. Through the adjustment of the protopolymer gel formulation, direct control of the Young’s modulus of the DPG particle systems was achieved. What is more, the mechanical strength of DPG particle systems has been quantitatively characterized for the first time by our group, which has realized the direct control of the Young’s modulus of DPG particle systems by adjusting the original formulas of bulk gels [38]. It was found that increase in the mechanical strength of a DPG particle system showed a positive effect on its regulation ability with respect to a reservoir [30]. However, there is a lack of systematic research on the effect of reservoir conditions on the mechanical strength of DPGs, as well as the desired range of mechanical strength for optimum reservoir regulation performance.

In this paper, DPG particle systems with different Young’s moduli were prepared by physical shearing of bulk gels with different formulas (different concentrations of polymers and cross-linkers). The influence of Young’s modulus on the reservoir regulation effects of the DPG particle systems, including migration performance, profile control capacity and enhanced oil recovery ability, were explored. The desired Young’s modulus range and applicable conditions for reservoir regulation were optimized. The effects of reservoir temperature and salinity conditions on the Young’s moduli of the DPG particles systems were also studied, providing basic theoretical guidance for the application of DPG particle systems in efficient oilfield development.

## 2. Results and Discussion

### 2.1. The Influence of Young’s Modulus on the DPG Particle System Reservoir Regulation Effects

**Figure 1:**
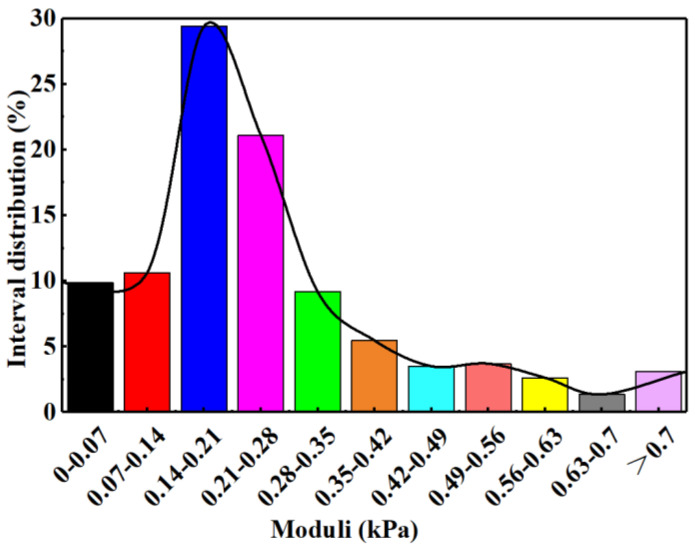
The interval distribution of the Young’s moduli of a representative DPG particle system (with 0.3% polymer + 0.8% cross-linker in this case).

The mechanical strengths of six DPG particles were calculated by fitting lots of force curves with the JKR theoretical equation. The Young’s modulus values of the DPG particle systems were not fixed values but presented columnar distributions. For example, the interval distribution of the Young’s moduli of a representative DPG particle system with 0.3% polymer and 0.8% cross-linker is shown in Figure 1. The Young’s modulus value and its percentage were multiplied, then these products were summed. Table 1 illustrates the weighted average Young’s modulus corresponding to each original formula.

**Figure 2:**
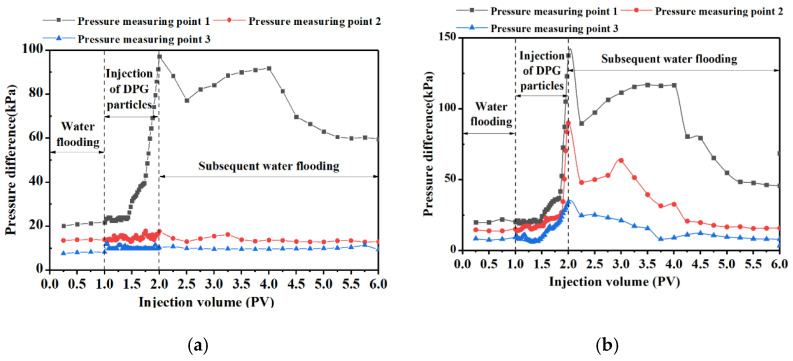

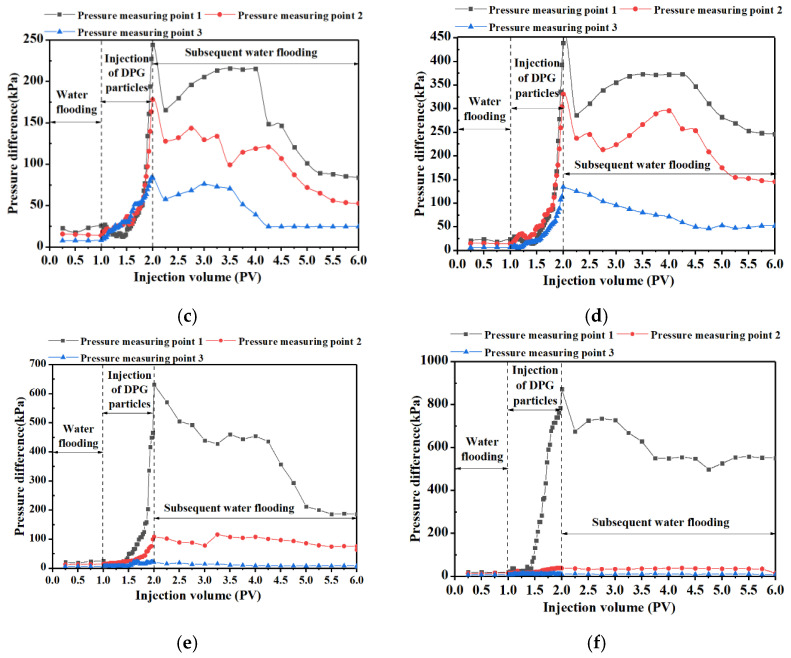
Migration performances of DPG particle systems with different Young’s moduli. (**a**) 0.2% polymer + 0.6% cross-linker, E = 0.082 kPa. (**b**) 0.25% polymer + 0.7% cross-linker, E = 0.19 kPa. (**c**) 0.3% polymer + 0.8% cross-linker, E = 0.257 kPa. (**d**) 0.35% polymer + 0.9% cross-linker, E = 0.762 kPa. (**e**) 0.4% polymer + 1.0% cross-linker, E = 1.222 kPa. (**f**) 0.45% polymer + 1.1% cross-linker, E = 1.723 kPa.

As the particle sizes of the DPG particles prepared under suitable shearing rates and times were similar, the differences in the migration performances of the DPG systems were mainly reflected in the differences in Young’s moduli. DPG particles were injected into the simulation cores, and the changes in pressure at each measuring point for the six systems are shown in Figure 2. As shown in Figure 2a, when the Young’s modulus of the DPG particle system was low (0.082 kPa), the pressure difference at both pressure measuring points 2 and 3 rose very little. The volume of the produced fluid was almost the same as that of the injected pore. The system with a Young’s modulus of 0.082 kPa was easily deformed, and most of the particles passed through the pore throats directly.

Figure 2b–d shows the variation in the displacing pressure differences when injecting DPG particle systems with Young’s moduli in the range of 0.19–0.762 kPa. As the Young’s moduli of the DPG particle systems increased to 0.19 kPa (Figure 2b), the displacing pressure difference at each measuring point increased to a certain extent. With the continuous increase in the Young’s moduli of the DPG particle systems, the pressure differences at each pressure measuring point increased relatively. Moreover, with the increase in the distance between the pressure measuring point and the injection end, the increase range of the displacing pressure difference at the corresponding pressure measuring point decreased. Therefore, it was concluded that the system did not aggregate at the injection end when the average Young’s moduli of the DPG particle systems were in the range of 0.19–0.762 kPa. In these systems, some particles with a higher Young’s modulus preferentially entered the large pore throats, and the accumulation was retained in the porous media to bridge and plug, improving the flow resistance [25]. Simultaneously, some soft DPG particles of these systems could enter the deep formation through elastic deformation due to lower Young’s moduli, thus realizing the role of deep migration and deep regulation [39].

The migration performances of the DPG particle systems with relatively high Young’s moduli are shown in Figure 2e,f. When the DPG particle system with an average Young’s modulus of 1.222 kPa was injected, the pressure at measuring point 2 still rose slightly, while the pressure at measuring point 3 remained almost unchanged. These results indicated that when the Young’s modulus was 1.22 kPa, the DPG system particles still had a certain deformation ability, and there was no aggregation of a large number of particles at the injection end. However, only a small number of particles could still pass through the pore throats, and the deep regulation ability of the DPG particles began to show deficiencies. As the Young’s modulus of the DPG particle system continued to increase to 1.723 kPa, the displacing pressure differences at both measuring points 2 and 3 no longer rose significantly. The particles in this system had poor deformability and mainly aggregated at the injection end, leading to the difficulties in achieving deep migration.

The residual resistance coefficient values in Table 2 could reflect whether the DPG particles were retained after subsequent water flooding with multiple pore volumes. When the average Young’s modulus of the system was 0.082 kPa, it was difficult for the too soft DPG particle system to form an effective accumulation in the porous medium. The increase in the Young’s modulus of DPG particle system would weaken its deformation ability, but the increase in the modulus did not directly affect its migration performance negatively.

When the Young’s modulus of the DPG particle system reached 0.19 kPa, the particles began to exhibit an elastic deformation–deformation recovery ability that was conducive to deep migration, so that effective plugging effects were exerted in all areas of the simulated core. When the average Young’s modulus of the DPG particle system was between 0.257 and 0.762 kPa, the plugging rate at each pressure measuring point could reach more than 90%.

In comparison with conventional Polyacrylamide gel plugging agents of the same type [40,41,42,43,44], DPGs exhibit superior plugging efficiency and still retain a certain plugging ability at a distance from the injection well. The cost-effectiveness analysis suggests that DPGs hold considerable potential for practical applications.

Meanwhile, the residual resistance coefficient values were always high after subsequent water flooding with multiple pore volumes, indicating that the system still had good mechanical strength and that the particles were retained to form physical barriers.

As the average Young’s moduli of the DPG particles increased to greater than 1.222 kPa, the action distance was relatively short but the systems still had a certain migration ability. In addition, the DPG particles with Young’s moduli higher than 1.222 kPa consumed more energy when elastically deformed by external forces, such as the extrusion of the pore throat wall, and the ability to recover from deformation weakened. During the process of subsequent water flooding, the bridging and accumulation between particles were flushed to be dispersive, resulting in a reduction in residual resistance coefficient values. The DPG particle systems with too high Young’s moduli mainly plugged in the near-wellbore zone, which made it difficult to meet the requirements of deep control and flooding in engineering practice.

**Figure 3:**
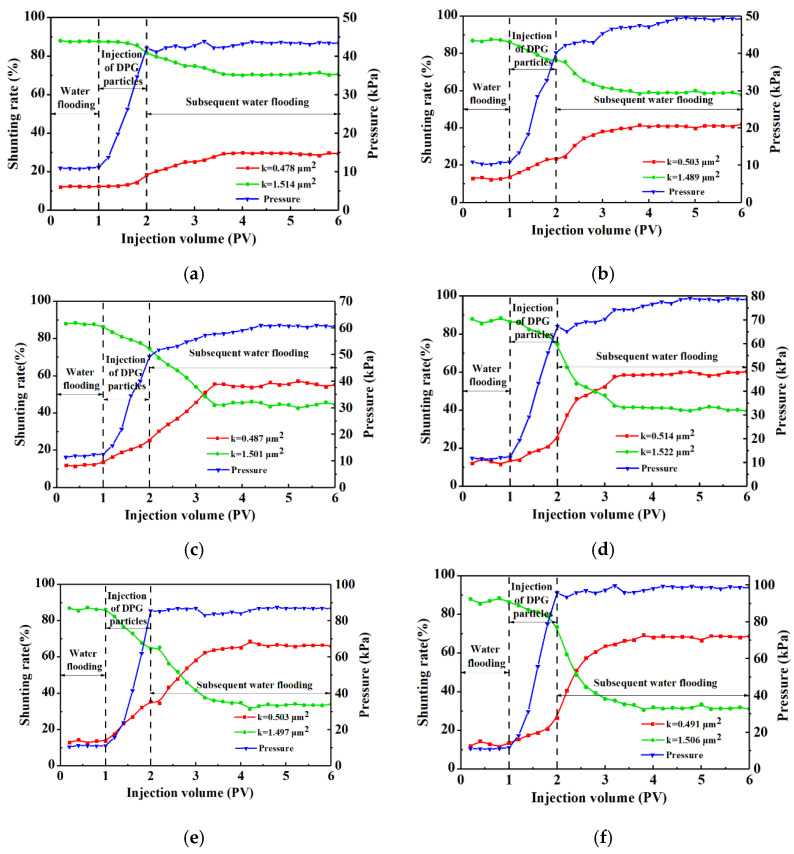
Profile control capacities of DPG particle systems with different Young’s moduli. (**a**) 0.2% polymer + 0.6% cross-linker, E = 0.082 kPa. (**b**) 0.25% polymer + 0.7% cross-linker, E = 0.19 kPa. (**c**) 0.3% polymer + 0.8% cross-linker, E = 0.257 kPa. (**d**) 0.35% polymer + 0.9% cross-linker, E = 0.762 kPa. (**e**) 0.4% polymer + 1.0% cross-linker, E = 1.222 kPa. (**f**) 0.45% polymer + 1.1% cross-linker, E = 1.723 kPa.

The heterogeneity of the double-layer profiles was adjusted by DPG particle systems with different Young’s moduli, and the variation in the pressure and shunting rate of two sand-packed pipes is shown in Figure 3. In Figure 3a,b, the Young’s moduli of DPG particle systems were relatively low (≤0.19 kPa). In these kinds of systems, particles with larger Young’s moduli directly enter the high-permeability zone and plugging occurs. At the same time, some soft particles with extremely small moduli entered the low-permeability layer. Therefore, the subsequently injected fluids flowed in both high- and low-permeability channels. The sand-packed pipe with higher permeability still had a high shunting rate, making the profile improvement effect not significant enough.

As illustrated in Figure 3c, the injection of a DPG particle system with a Young’s modulus of 0.257 kPa distinctly increased the shunting rate of the low-permeability sand-packed pipe in the subsequent water flooding process to more than 50%. These results could be explained by the fact that the DPG particle system with a Young’s modulus of 0.257 kPa gradually had a slightly better elasticity, which was beneficial to the adjustment of the seepage profile. The deformation recovery ability of the DPG particle system led to the denser bridging accumulation formed in the high-permeability layer, and the liquid production of the high-permeability sand-packed pipe decreased, thus improving the profile improvement rate distinctly. The profile improvement rate was increased distinctly from 67.2% to 89.02%

Figure 3c–f show that the injection pressure rose significantly as the Young’s moduli of the DPG particle systems continued to increase. These results indicate that the particles preferentially migrate into the high-permeability layer and form effective plugging inside. For the DPG particle systems with a Young’s modulus exceeding 0.257 kPa, there were hardly any very small values in the Young’s modulus distributions for the systems. The accumulation of particles in the high-permeability sand-packed pipe could force the subsequent flow to divert into small pores in the low-permeability reservoir. The increase in the modulus of the injected DPG particles caused the system to almost no longer pollute the low-permeability layer, so that the shunting fluid capacity of the low-permeability layer was gradually better than that of the high-permeability layer. For the system employing DPGs with a Young’s modulus exceeding 0.762 kPa, there was a significant increase in injection pressure, resulting in a final shunting rate of 93.84% for the heterogeneous rock core profile, which surpassed the performance of current conventional Polyacrylamide gel plugging agents [45,46]. However, plugging agents, such as industrial gels and polymer microspheres, have poor shear resistance and injection ability, resulting in complete plugging near the injection well, which greatly reduces the conformance control effect when the injected water reflows near the wellbore [47,48,49]. In contrast, conformance control of the reservoir is achieved by adjusting the Young’s modulus of DPGs, allowing them to migrate deep into the high-permeability reservoir and improve the formation heterogeneity [22,50].

It could be concluded that in the Young’s modulus range of 0.082–1.723 kPa, the ability of the DPG particle systems to improve the profile heterogeneity was enhanced with modulus increase. DPG particle systems with higher Young’s moduli showed more advantages in microscopically expanding the sweep coefficient and macroscopically improving the heterogeneity of the reservoir. Injecting a DPG particle system with a relatively high Young’s modulus was beneficial in reducing the damage to the non-target layer, so as to achieve the goal of deep regulation in the reservoir.

**Figure 4:**
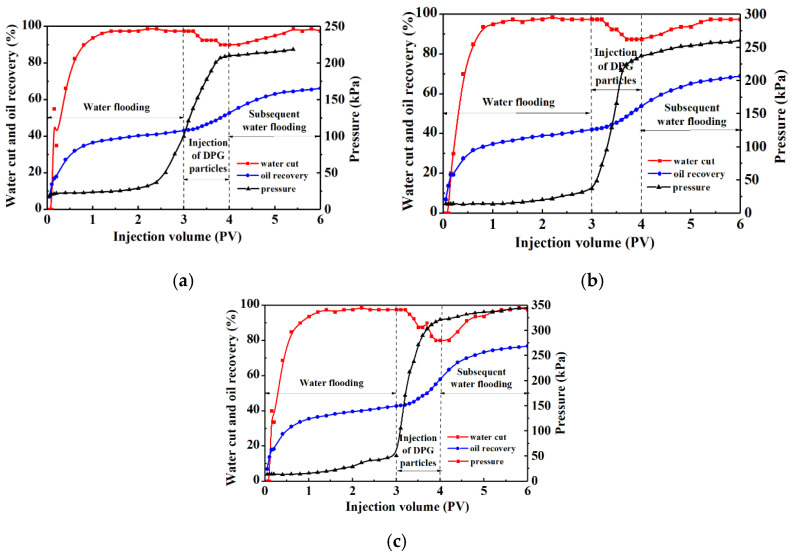
Enhanced oil recovery abilities of DPG particles with different Young’s moduli. (**a**) 0.25% polymer + 0.7% cross-linker, E = 0.19 kPa. (**b**) 0.3% polymer + 0.8% cross-linker, E = 0.257 kPa. (**c**) 0.35% polymer + 0.9% cross-linker, E = 0.762 kPa.

Figure 4 shows the effect of Young’s modulus on the enhanced oil recovery ability of the DPG particle systems. The mechanical strength was required to not only guarantee the deep migration performance of the particles, but also ensure that the systems could obviously improve the heterogeneity of the profile. Therefore, the systems with suitable Young’s modulus values ranging from 0.257 to 0.762 kPa were chosen to carry out the experiments.

The pressure increased significantly during the process of injecting the DPG particle systems, indicating that the particles effectively plugged large pores through their deformation and migration, and the particles were adsorbed and retained in the dominant channels. The above indicates that the DPGs have good injectability and deep migration ability only when their Young’s modulus values exceed 0.19 kPa. When the Young’s modulus of DPG particle systems increased from 0.257 kPa to 0.762 kPa, it was easier for particles to pack densely in the high-permeability layers. Under the influence of the difference between the surface polarity of the DPG particle systems and that of the residual oil, the injection of the DPG particle systems with higher Young’s moduli could make the water cut drop more obviously. Therefore, the subsequently injected fluids were diverted to the low-permeability zones with high oil saturation, thus sweeping and displacing out the residual oil. The injection pressure of the subsequent water drive increased accordingly. It can be observed from Figure 4 that the water flooding recovery after DPG injection was over 20% higher than that based on the primary water flooding foundation, surpassing that of conventional gel plugging agents [28,51].

Based on this study and previous DPG reports, considering the material costs, applying DPG particles with moduli within the range of 0.19–0.297 kPa (polymer concentration: 0.25–0.4%; cross-linker concentration: 0.7–0.9%) would provide optimum reservoir control performance.

### 2.2. The Effects of Reservoir Conditions (Temperature and Salinity) on the Young’s Moduli of the DPG Particle Systems

In a long-term construction process, the reservoir environment would change the Young’s modulus of a DPG particle system, which would affect the elastic deformation ability of the particles in the formation. As a result, a representative DPG particle system (0.3% polymer and 0.8% cross-linker) was aged at different temperatures and salinity conditions to observe the patterns of change in Young’s modulus values.

**Figure 5:**
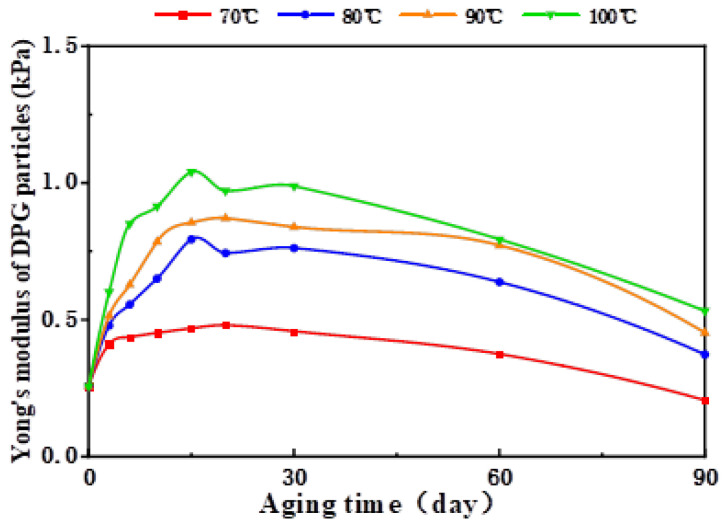
Patterns of change in the Young’s modulus of the DPG particle system at different temperatures.

Figure 5 shows the overall variation in the Young’s modulus of this typical DPG particle system with aging temperature. The higher the temperature, the higher the Young’s modulus peak value that the DPG particle system could reach, and the higher the modulus value after 90 days of aging. It could be that the increase in temperature promoted the Brownian motion of the DPG particles, weakening the hydrogen bond interactions and the electrostatic interactions between the DPG particles, resulting in a relatively large change in the Young’s modulus of the system when the temperature was raised [52,53,54]. After a period of aging, the aggregation morphology between particles was no longer tight. Under the action of a certain external force, a small amount of free water entered the system, which reduced the Young’s modulus of the DPG particle system.

However, the DPG particle system could always maintain good mechanical strength under medium- and high-temperature conditions. The Young’s modulus values of this DPG particle system after 90 days of aging at 70–100 °C showed only slight fluctuations compared with the initial modulus value. Owing to the close relationship between the ability of the DPG particle system to retain water and its structure, the stable structure meant that the DPG particles were less prone to water loss. The dense spatial network structure formed by the polymer and the cross-linker ensured the bulk gel had strong thermal stability, so that the structure of the DPG particle system prepared by physical shearing was not easily damaged. Therefore, this DPG particle system could meet the requirements of deep control and flooding of medium- and high-temperature oilfields with formation temperatures of 70 to 100 °C.

**Figure 6:**
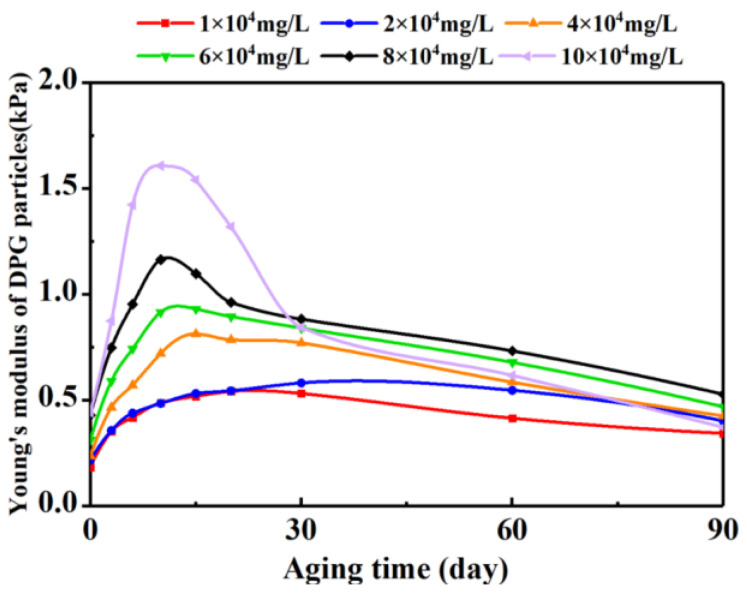
Patterns of change in the Young’s moduli of the DPG particle systems under different salinity conditions.

The effect of salinity on the Young’s modulus of the DPG particle system during the aging process is shown in Figure 6. When this representative system was aged under low-salinity conditions, the probability of the appearance of particles with stronger hardness was low. It could be that the surface of the DPG particle system was negatively charged and that some metal salt ions neutralized these negative charges. In an environment with lower salinity, salt ions could be adsorbed on the surface of DPG particles, which could reduce the effect of electrostatic repulsion to ensure the stability of particles. Therefore, in low-salinity reservoirs, the change in the Young’s modulus of the DPG particle system was very small given the prolongation of aging time.

As the salinity continued to increase, the DPG particle system might have undergone an oxidation reaction in the formation. At the same time, salt ions could play a stronger electrostatic shielding effect and salt bridging effect, resulting in reduction in the support force of the pore throat wall on the particles and the frictional resistance between the pore throat wall and the particles of the DPG particle system [55,56]. The DPG particles reduced the binding effect on water molecules; hence, the hardness of the particles became stronger. The Young’s modulus of the DPG particle system greatly increased in the early stage of aging, the energy consumed when deformation occurred under the action of external force increased, and the deformation ability was relatively weakened, while, when this system was aged in a high-salinity environment of 10 × 10^4^ mg·L^−1^, the mutual slip between the DPG particles was more easily affected by friction under the influence of the size effect, resulting in an augmentation of the amplitude of increase in the Young’s modulus. After the Young’s modulus reached the peak value, the amount of charge carried by the DPG particle system changed with the salinity of the formation water, which caused the expansion of the system. Then, the Young’s modulus of the DPG particle system decreased sharply. However, when the salinity was between 1 and 10 × 10^4^ mg·L^−1^, the value of the Young’s modulus of the DPG particle system after 90 days of aging was always higher than or close to the initial value.

With increase in temperature or salinity, the Young’s modulus of the DPG particle systems also increased moderately. At the same salinity (4.56 × 10^4^ mg/L), in the temperature range of 70–100 °C or at the same temperature (90 °C), within the salinity range of 1–4 × 10^4^ mg·L^−1^, when the DPG particle systems were aged for 90 days, the Young’s modulus was consistently quantified to be between 0.19 and 1.222 kPa. Particles in this Young’s modulus range could smoothly migrate to the deep formation to achieve the purpose of improving profile heterogeneity and enhancing oil recovery. It was seen from the above experiments that the reservoir regulation abilities of the DPG particles were positively correlated with the Young’s modulus. Consequently, it was fully demonstrated that the performances of the DPG particle systems were not directly deteriorated after injection into the formation. A moderate increase in temperature or salt ion concentration could have improved the carrying capacity of the DPG particle systems after the particles were connected to each other, which had a favorable impact on the reservoir regulation abilities of the DPG particle systems. The high-salinity condition caused the DPG particles to show a tendency to spontaneous aggregation as soon as they entered the formation, weakening their deep migration ability. However, due to the wide distribution range of the Young’s moduli of the particles, there were also particles with small modulus values in the system after aggregation, and the subsequent injection of fluid could still promote the action distance of the DPG particles.

## 3. Conclusions

In this study, the influence of Young’s modulus on the reservoir regulation effects of DPG particles, including migration performance, profile control capacity and enhanced oil recovery ability, were explored. Within the modulus range of 0.082–1.723 kPa, DPG particles exhibited excellent injection performance. An increase in the Young’s modulus of DPGs results in a significant increase in injection pressure, leading to the preferential migration of particles to high-permeability reservoirs, resulting in effective plugging. The shunting rate for the heterogeneous rock core profile is more than 90% for DPG systems with Young’s moduli greater than 0.762 kPa. However, only the DPG particles with a modulus range of 0.19–0.762 kPa could achieve both adequate blockage in large pore throats and also migration to deep reservoirs through deformation. Otherwise, the injection of DPGs on the basis of primary water drive resulted in an increase of more than 20% in water drive recovery, demonstrating that the DPGs showed enhanced performance in improving the heterogeneous profile as well as oil recovery ratio with the increase in Young’s modulus. Therefore, combined with the cost, the desired Young’s modulus range for reservoir regulation was optimized. In addition, the effects of reservoir temperature and salinity conditions on the Young’s moduli of the DPG particle systems were also studied. These results indicated that the practical reservoir regulation performances of DPGs can be improved by adjusting the mechanical strength, and the application effect of DPGs can also be enhanced by moderate increases in temperature and salinity, providing basic theoretical guidance for the application of DPGs in efficient oilfield development.

## 4. Materials and Methods

### 4.1. Materials and Devices

Shanghai Macklin Biochemical Co., Ltd. provided the dry powder of the polyacrylamide and phenolic resin cross-linker; dry powder of polyacrylamide with an average molecular weight of 14,000,000 was used to prepare the polymer solutions.

A colloidal mill (CM-2000 type, Shanghai Yiken Instruments, Ltd., Shanghai, China) was used to physically shear bulk gels into DPG particles. An atomic force microscope (AFM; Multimode 8, Bruker, Mannheim, Germany) was used to measure the Young’s moduli of DPG particles.

### 4.2. Preparation and Characterization of DPG Particles with Different Mechanical Strengths

Polymer solutions were mixed with phenolic resin cross-linkers to form uniform gel solutions. After reaction at 90 °C for 24 h in an oven, bulk gel systems were produced. By controlling the rotation speed and the minute of cyclic shearing of the colloidal mill, DPG particle systems with similar particle sizes but different mechanical strengths were obtained.

A spherical silica particle with a diameter of 5 μm was stuck to the original tip of the SCANASYST-FLUID probe of the AFM. The radius of the modified probe was *R_tip_*. The force–distance curves of the center pixels of the spherical DPG particles with a radius of *R_s_* and a Poisson ratio of *v* were recorded in a liquid environment using PeakForce mode [57]. By fitting these force curves with the JKR model, the Young’s modulus *E* values of the DPG particle systems were calculated using the following equations [35,58,59,60].

(1) $$R=\frac{R_{\mathrm{tip}}{\cdot R}_{s}}{R_{\mathrm{tip}}{+R}_{s}}$$

(2) $$E=-\frac{{3(1-v}^{2})}{4}{\left(\frac{1+\sqrt[{3}]{16}}{3}\right)}^{\frac{3}{2}}\cdot \frac{F_{1}}{\sqrt{{R\delta }_{s}{}^{3}}}$$

where *R* is the equivalent radius of the probe and the sample μm, *F*_1_ is the maximum adhesion force between the probe and the sample nN, and *δ_s_* is the distance between the probe and the sample.

### 4.3. Variation in Migration Performance of the DPG Particles with Young’s Modulus

**Figure 7:**
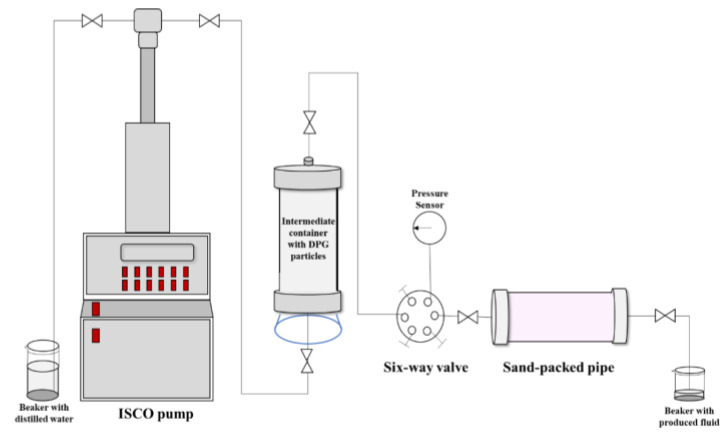
Illustration of the multi-point pressure test setup.

The experimental device was depicted in Figure 7. The Φ25 mm × 300 mm multi-point pressure test sand-packed pipe was selected as the simulation core. The initial permeability kw_0_ was kept unchanged at about 0.5 μm^2^, and its value was measured by a water flooding experiment. After injecting DPG particles with different Young’s moduli, the sand-packed pipes were placed in an oven set at 90 °C for aging for 14 days. Then, the subsequent water flooding was carried out until the pressure at each measuring point of the simulated core became stable.

The permeability of the simulated core during subsequent water flooding was recorded as *k_w_*_2_. Plugging rates (*R_p_*) at each measuring point were calculated using the following equations, where *F_rr_* was the residual resistance factor [61,62]. The migration performances of the DPG particles could be reflected in *R_p_* and *F_rr_*.

(3) $$k\;=\;\frac{Q\mu L}{A\Delta p}$$

(4) $$R_{p}=\frac{k_{w_{0}}{-k}_{w_{1}}}{k_{w_{0}}}$$

(5) $$F_{\mathrm{rr}}=\frac{k_{w_{0}}}{k_{w_{2}}}$$

### 4.4. Variation in Profile Control Capacity of the DPG Particles with Young’s Modulus

**Figure 8:**
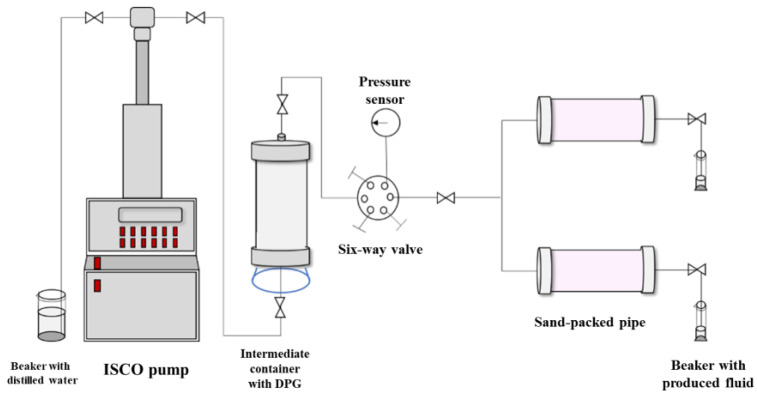
Section homogenization performance test device diagram.

The experimental device was depicted in Figure 8. Two Φ25 mm × 200 mm sand-packed pipes were paralleled to simulate double-layer heterogeneous cores. The initial permeabilities of the high- and low-permeability sand-packed pipes were 1.5 μm^2^ and 0.5 μm^2^, respectively. The water-absorption rates of the high- and low-permeability pipes before profile control were *Q_hb_* and *Q_lb_*, respectively. DPG particles were injected into the simulation double-layer cores with similar permeability contrasts of 1:3 at a constant speed of 0.5 mL/min.

The sand-packed pipes injected with DPG particles were aged for 14 days. Then, the subsequent water flooding was carried out until the pressure at the output end of the simulated core became stable. The water-absorption rates of the high- and low-permeability pipes during subsequent water flooding were *Q_la_* and *Q_ha_*_,_ respectively. The shunting rate *f*, which represented the profile improvement capacity of the system, was obtained using the following equation.

(6) $$f=(1-\frac{Q_{h_{a}}}{Q_{l_{a}}}/\frac{Q_{h_{b}}}{Q_{l_{b}}})\;\times \;100\%$$

### 4.5. Variation in Enhanced Oil Recovery Ability of the DPG Particles with Young’s Modulus

Considering the modulus range of DPG particles with good migration performances and profile control capacities, the effect of Young’s modulus on increasing the oil production ability of DPG particles was investigated. The simulated cores with similar permeabilities of about 0.5 μm^2^ were saturated with crude oil and then aged in an oven for 1 day. After water flooding until the water cut of the core reached 98%, 1 pore volume DPG particles were injected into the simulated core at a rate of 0.5 mL/min.

The simulated cores were aged for 14 days, and then the subsequent water flooding was carried out until the water cut reached 98% again. During the experiment, the liquid production, oil production and pressure values were continuously recorded at 0.2 PV per injection, and the variation laws for water cut and oil recovery were calculated. The above experiments that evaluated the reservoir regulation abilities of the DPG particles were all conducted at 90 °C.

### 4.6. The Effect of Reservoir Environment on the Young’s Modulus of the DPG Particles

A DPG particle system from the bulk gel formulated with 0.3% polymer and 0.8% cross-linker was chosen as a typical representative system to analyze its temperature and salt-resistance properties. When investigating the effect of temperature on the Young’s modulus of the DPG particles, the system was aged at 70–100 °C for 90 days with constant salinity (4.56 × 10^4^ mg·L^−1^). Then, the temperature was fixed at 90 °C, and the bulk gel systems were mixed with simulated formation water with different salinity conditions of 1–10 × 10^4^ mg·L^−1^ to prepare DPG particles. The effect of salinity on Young’s modulus during the aging process was explored.

## Figures and Tables

**Table 1:** Weighted average Young’s modulus values corresponding to each original formula.

Formula of Bulk Gels	E of DPG Particle System, kPa
0.2% polymer + 0.6% cross-linker	0.082
0.25% polymer + 0.7% cross-linker	0.19
0.3% polymer + 0.8% cross-linker	0.257
0.35% polymer + 0.9% cross-linker	0.762
0.4% polymer + 1.0% cross-linker	1.222
0.45% polymer + 1.1% cross-linker	1.723

**Table 2:** The deep regulation ability of DPG particle systems with different Young’s moduli.

Formula	E of DPG Particles System/kPa	Plugging Rate			Residual Resistance Coefficient Value		
		Measuring Point 1	Measuring Point 2	Measuring Point 1	Measuring Point 1	Measuring Point 2	Measuring Point 3
0.2% polymer + 0.6% cross-linker	0.082	79.2	23.47	25.68	2.95	1.11	1.23
0.25% polymer + 0.7% cross-linker	0.19	85.51	83.74	75.42	2.29	1.08	1.08
0.3% polymer + 0.8% cross-linker	0.257	90.57	91.06	90.59	3.66	3.31	3.16
0.35% polymer + 0.9% cross-linker	0.762	95.35	95.33	95.28	12.07	9.41	8.21
0.4% polymer + 1.0% cross-linker	1.222	96.67	87.79	44.71	8.88	5.71	1.29
0.45% polymer + 1.1% cross-linker	1.723	97.66	62.11	28.06	27.03	1.04	1.19

## Data Availability

No new data were created or analyzed in this study. Data sharing is not applicable to this article.
